# Application value of ^18^F-FDG PET/CT in primary spleen angiosarcoma with liver metastasis: a case report and literature review

**DOI:** 10.3389/fonc.2024.1366560

**Published:** 2024-05-24

**Authors:** Mingyan Shao, Wanling Qi, Rong Xu, Zhehuang Luo, Fengxiang Liao, Sisi Fan

**Affiliations:** ^1^ Department of Nuclear Medicine, Jiangxi Provincial People’s Hospital, The First Affiliated Hospital of Nanchang Medical College, Nanchang, Jiangxi, China; ^2^ Department of Pathology, Jiangxi Provincial People’s Hospital, The First Affiliated Hospital of Nanchang Medical College, Nanchang, Jiangxi, China

**Keywords:** primary, spleen angiosarcoma, liver metastasis, CT, ^18^F-FDG PET/CT

## Abstract

**Background:**

Primary splenic angiosarcoma (PSA) is a rare neoplasm. It is a malignant tumor derived from endothelial cells of the splenic sinuses. PSA has an unknown etiology, a high degree of malignancy, easy early metastasis, atypical clinical symptoms and imaging findings, and difficult early diagnosis. This paper reports the ^18^F-FDG PET/CT findings of a case of PSA with intrahepatic metastasis; summarizes its clinical, imaging, and pathological data; and reviews the literature.

**Case description:**

A 64-year-old male patient presented with left lower abdominal distending pain without obvious causes on 13 March 2022. The pain was persistent and dull and worsened after sitting and eating. Blood routine examination results were RBC ↓ 3.33 × 10^12^/L, WBC ↑ 12.32 × 10^9^/L, and PLT ↓ 40 × 10^9^/L. The tumor markers indicated CA125 ↑ 47.0 U/ml, AFP (−), CEA (−), CA199 (−), and CA724 (−). Non-contrast-enhanced CT scan of the abdomen showed that the spleen was significantly enlarged in volume and irregular in shape and had multiple nodules and clumpy low-density shadows, unclear boundaries, uneven density, and multiple necrotic areas. Enhanced CT showed diffuse uneven mild enhancement of the spleen, and the degree of enhancement increased with time. Multiple nodular low-density shadows were seen in the liver, which were slightly enhanced by the enhanced scan.^18^F-FDG PET/CT showed multiple nodular and massive lesions in the spleen with multiple necrotic areas. There were multiple nodular lesions in the liver, the level of FDG metabolism increased, the SUVmax of the spleen lesions was 9.0, and the SUVmax of the liver lesions was 5.6. The ^18^F-FDG PET/CT diagnosis was splenic malignancy with liver metastasis. Finally, after a multidisciplinary discussion, it was decided to perform laparoscopic total splenectomy and portal vein infusion chemotherapy. Pathological examination showed that the tumor cells were round, oval, or fusiform, with obvious atypia, arranged into a cable or anastomosed vascular lumen. The final diagnosis was primary splenic angiosarcoma with massive necrosis. After surgery, the patient received antitumor combined therapy and died 5 months later.

**Conclusion:**

The incidence of PSA is very low, and its clinical and radiological manifestations lack specificity. ^18^F-FDG PET/CT imaging has a certain diagnostic value for PSA and significant utility in preoperative staging, guiding biopsy procedures, evaluating postoperative treatment response, and monitoring disease recurrence. PSA should be considered in the presence of a space-occupying lesion within the spleen that exhibits necrotic areas, shows progressive enhancement on contrast-enhanced scans, and demonstrates heterogeneous increases in FDG uptake.

## Introduction

Primary splenic angiosarcoma (PSA) is a rare and highly malignant tumor that originates from endothelial cells of the splenic sinuses (1). It has a propensity for early metastasis, commonly affecting the liver, lungs, lymph nodes, and bone. The etiology of PSA remains unclear, and its clinical symptoms and imaging findings are often atypical, making early diagnosis challenging. Confirmation of PSA ultimately relies on pathological examination (2). To date, there are limited reports on the imaging manifestations of PSA, with most studies being case reports and even fewer focusing on the ^18^F-FDG PET/CT imaging characteristics. This paper reports the ^18^F-FDG PET/CT imaging findings in a case of PSA with liver metastasis. It aims to summarize the clinical, imaging, and pathological data; review the relevant literature; and enhance our understanding and diagnostic capabilities for this disease. The findings may also provide valuable insights for the management of future patients.

## Case description

**Figure 1:**
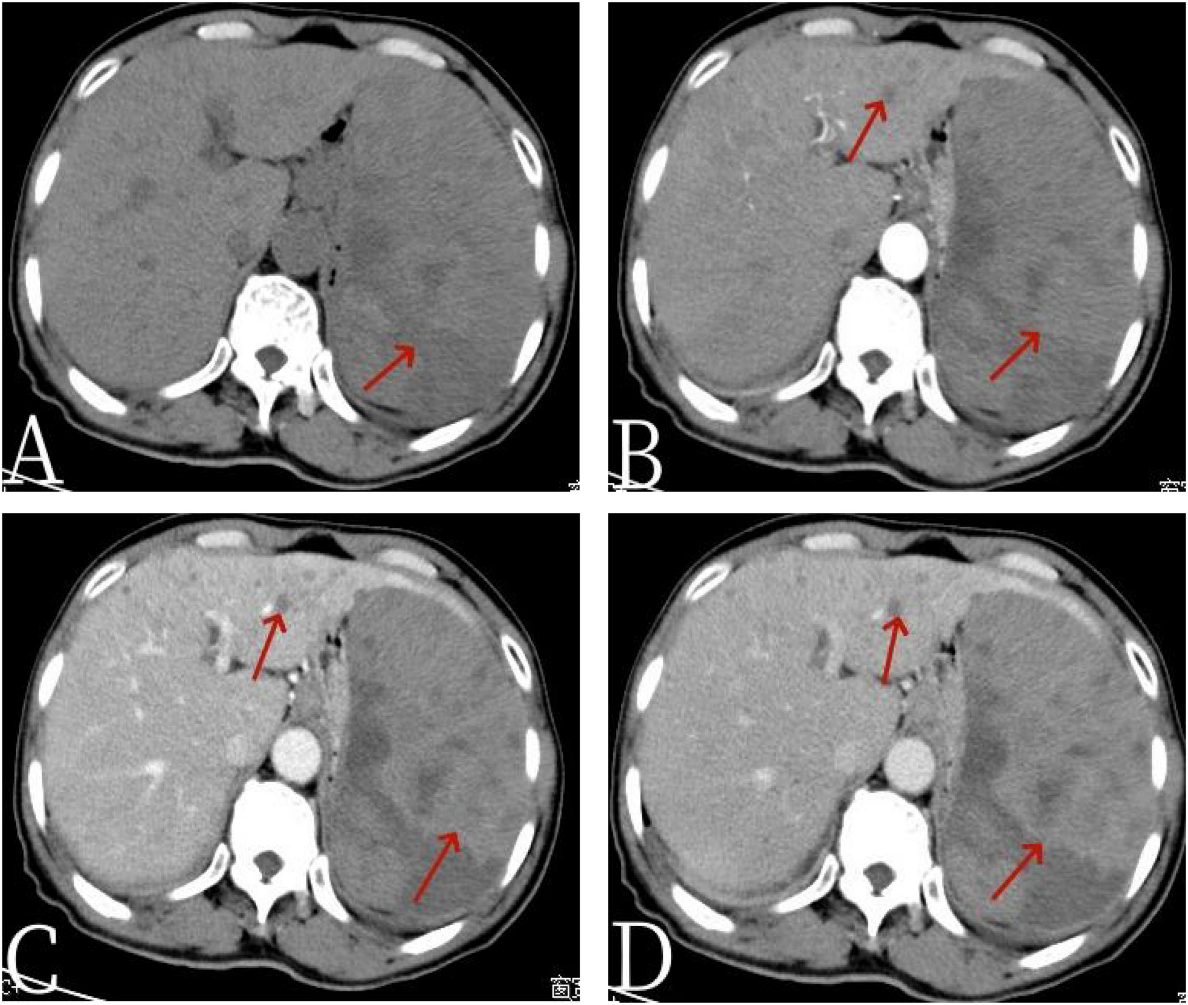
The 67-year-old male patient with primary splenic angiosarcoma. **(A)** Non-contrast-enhanced CT, **(B)** enhanced CT-arterial phase, **(C)** enhanced CT-venous phase, and **(D)** enhanced CT-delayed phase. The non-contrast-enhanced CT scan of the abdomen revealed a spleen that was significantly enlarged in volume, with an irregular shape and the presence of multiple nodules and clumpy low-density shadows. The boundaries of these lesions were unclear, and the density throughout the spleen was uneven, indicating multiple areas of necrosis [arrow, **(A)**]. In the enhanced CT images, there was diffuse, uneven mild enhancement of the spleen, with the enhancement becoming more pronounced over time [arrows, **(D)**]. Additionally, the scan identified multiple nodular low-density shadows in the liver [arrows, **(B)**], which showed slight enhancement upon closer examination.

**Figure 2:**
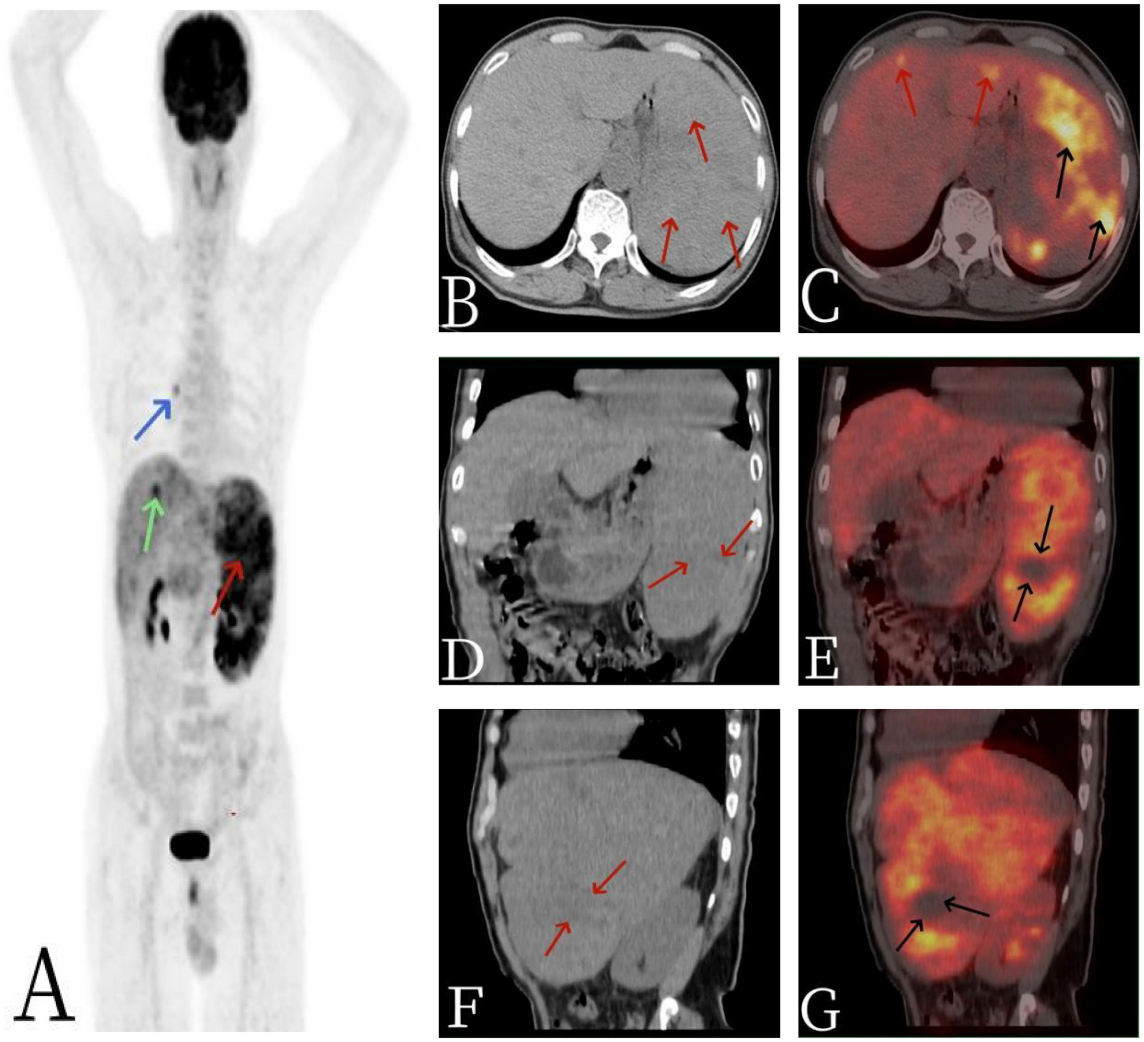
The 67-year-old male patient with primary splenic angiosarcoma. **(A)** Whole body MIP, **(B)** axial CT, **(C)** axial fusion, **(D)** coronal CT, **(E)** coronal fusion, **(F)** sagittal CT, and **(G)** sagittal fusion. The CT scan revealed multiple nodular and massive lesions within the spleen [arrows, **(B)**], accompanied by numerous necrotic areas [arrows, **(D, F)**]. ^18^F-FDG PET/CT imaging demonstrated an abnormal and uneven uptake of ^18^F-FDG throughout the spleen, with a maximum standardized uptake value (SUVmax) of 9.0 [black arrows, **(C)**], and no FDG uptake was observed in some of the necrotic regions [black arrows, **(E, G)**]. Additionally, there were multiple nodular lesions in the liver with elevated FDG metabolism, registering an SUVmax of 5.6 [red arrows, **(C)**]. A focal area of increased FDG uptake in the thorax was considered to be non-specific mediastinal lymph node activity [blue arrow, **(A)**].

**Figure 3:**
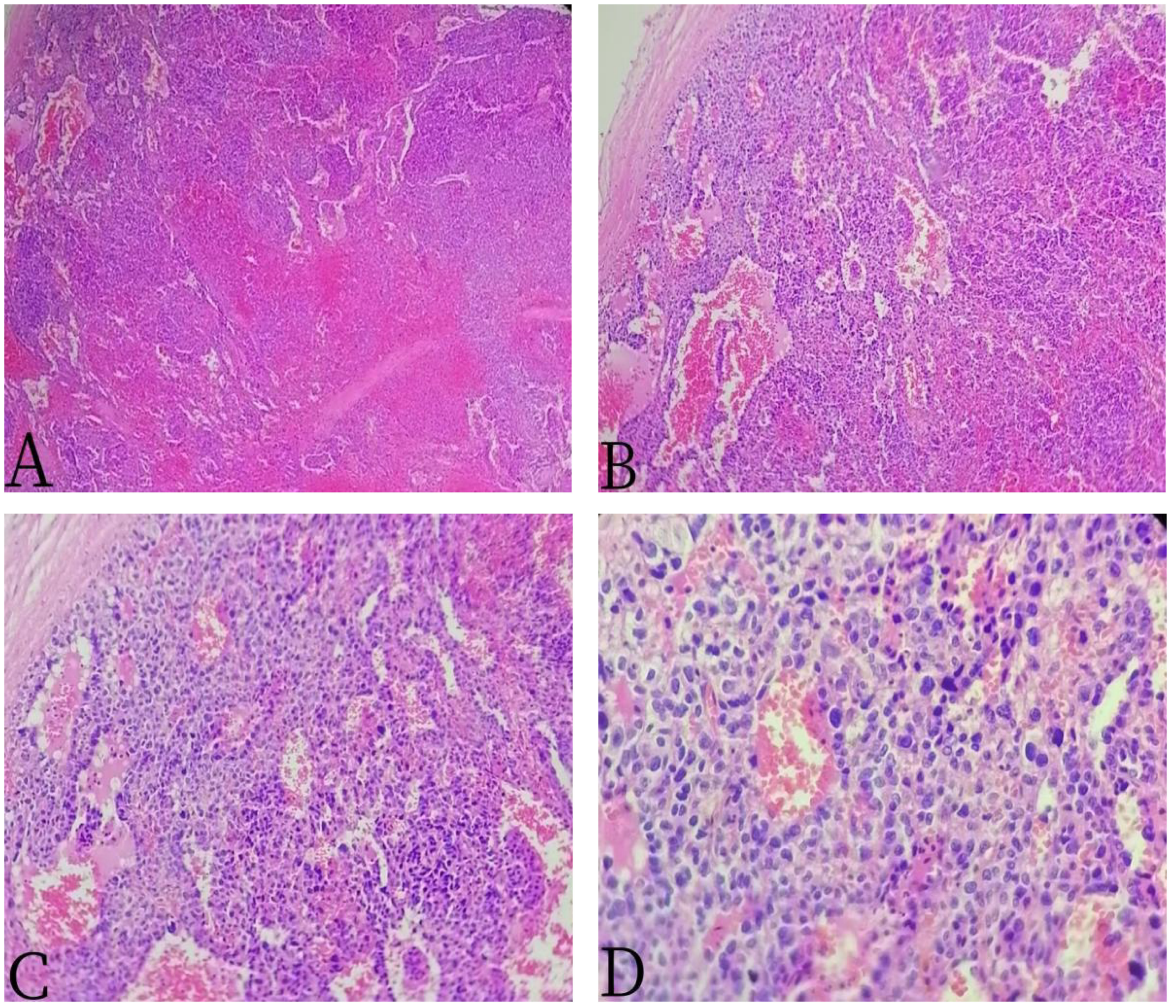
The 67-year-old male patient with primary splenic angiosarcoma. **(A)** H&E ×40, **(B)** H&E ×100, **(C)** H&E ×200, and **(D)** H&E ×400. The tumor cells were round, oval, or fusiform, with obvious atypia, arranged into a cable or anastomosed vascular lumen.

A 64-year-old male patient presented with unexplained distending pain in the left lower abdomen on 13 March 2022. The pain was persistent, dull, and exacerbated by sitting and after meals. There were no accompanying symptoms such as fever, cough, phlegm, nausea, vomiting, dizziness, headache, or diarrhea. Since the onset of symptoms, the patient had experienced poor appetite and approximately 5 kg of weight loss over the course of nearly a month. For further diagnosis and treatment, he was admitted to Jiangxi Provincial People’s Hospital. After admission, blood routine examination showed decreased red blood cell (RBC) count, 3.33 × 10^12^/L (3.5–5.5); elevated white blood cell (WBC) count, 12.32 × 10^9^/L (3–9); and reduced platelet (PLT) level, 40 × 10^9^/L (100–300). The tumor markers indicated CA125 ↑ 47.0 U/ml (0–35 U/ml), AFP (−), CEA (−), CA199 (−), and CA724 (−), and other laboratory tests were normal. The non-contrast-enhanced CT scan of the abdomen (**Figure 1**) revealed a significantly enlarged spleen with an irregular shape, multiple nodules, and clumpy low-density shadows. The boundaries were unclear, the density was uneven, and there were multiple areas of necrosis. The enhanced CT scan (**Figure 1**) showed diffuse, uneven mild enhancement of the spleen, with the degree of enhancement increasing over time. Multiple nodular low-density shadows were also observed in the liver, which showed slight enhancement on the enhanced scan. ^18^F-FDG PET/CT (**Figure 2**) revealed multiple nodular and massive lesions in the spleen with multiple necrotic areas. There were also multiple nodular lesions in the liver, with increased FDG metabolism. The maximum standardized uptake value (SUVmax) of the spleen lesions was 9.0, and the SUVmax of the liver lesions was 5.6. Due to the presence of multiple necrotic areas within the spleen and no surrounding lymphadenopathy, lymphoma was excluded by ^18^F-FDG PET/CT. The ^18^F-FDG PET/CT diagnosis was splenic malignancy with liver metastasis. Following a multidisciplinary discussion, it was decided to proceed with laparoscopic total splenectomy and portal vein infusion chemotherapy. The gross specimen consisted of one splenectomy specimen, a pile of grayish-red grayish-brown fragmented tissue measuring 10 × 19 × 8.5 cm. Pathological examination (**Figure 3**) revealed tumor cells that were round, oval, or fusiform with marked atypia, arranged in a cable-like pattern or forming vascular lumens. Immunohistochemistry showed CD31 (+), CD8 (+), P53 (+), CK (−), CD20 (−), CD3 (−), CD5 (−), PAX5 (−), CD4 (−), CD68 (−), CD45 (−), vimentin (+), SMA (−), D2–40 (−), S-100 (+), IgG (+), IgG4 (−), and Ki67 (+, approximately 70%). The final diagnosis was primary splenic angiosarcoma with massive necrosis. Following surgery, the patient underwent antitumor combination therapy. Unfortunately, he passed away 5 months later due to systemic progression of the disease compounded by drug toxicity.

## Discussion

PSA is a rare clinical condition, characterized by a malignant tumor that originates from endothelial cells lining the splenic sinuses (1, 2). First described by Langerhans in 1879, PSA has an annual incidence of approximately 0.14 to 0.25 per one million individuals, predominantly affecting the elderly with an average age of 50 to 60 years, and there is a slightly higher incidence in men than in women (10). The exact cause of PSA is not well understood but may be associated with exposure to radiation, chemotherapy, or substances such as thorium dioxide, arsenic, and peroxide (3). Clinical presentations of PSA are non-specific, often asymptomatic initially. As the disease progresses, most patients experience symptoms such as left upper abdominal pain, fever, splenomegaly, weight loss, and anemia. Laboratory findings commonly include elevated WBC counts, decreased RBC counts, and reduced PLT levels (4). The patient in this case presented with decreased RBCs, PLTs, and leukocytosis, which is consistent with the literature. In the advanced stages of the tumor, splenic enlargement can lead to rupture and bleeding, with approximately 25% of patients experiencing spleen rupture (5). In severe cases, this can result in hemorrhagic shock.

The diagnosis of PSA is primarily confirmed through pathology (6). Microscopically, the boundary between the tumor and the normal splenic tissue is indistinct, and the tumor exhibits an invasive growth pattern. The tumor cells appear oval, fusiform, and in some cases, epithelioid. They grow within blood sinusoids and fissures, displaying marked cellular atypia and frequent mitotic figures. The pathological manifestations of PSA can vary from case to case, generally presenting in four distinct forms (7–9): 1) the atypical proliferative or cellular arrangement of vascular endothelial cells; 2) tumor cells are arranged in a porous pattern; 3) malignant endothelial cells proliferate and form papillary lobules extending into the vascular and cystic spaces; and 4) flaky proliferating endothelial cells to polygonal tumor cells are arranged to form lesions. The immunohistochemical profile of PSA typically includes positivity for Vim (vimentin), CD34, CD31, and F8 (factor VIII) (11). Falk (12) and Neuhauser et al. (13) have suggested that the diagnosis of PSA should meet the following criteria: 1) at least two immunohistochemical indicators indicating vascular differentiation are positive, such as CD34, CD31, SMA, Vim, F8, UEA-1, CDl05, and VEGFR3. 2) At least one of the immunohistochemical test indicators indicating histophytocyte differentiation is positive, such as CD68, CD8, and S-100 proteins. In this case, CD31 (+), CD8 (+), P53 (+), and S-100 (+) in PSA were consistent with literature reports.

The imaging findings of PSA are often atypical. Ultrasonography typically reveals an enlarged spleen with uneven internal and external echoes and a broad, thick hypoechoic halo surrounding areas of strong echo. On CT scans, the main findings are splenic enlargement, which can manifest as single, multifocal, or diffuse lesions (14). These lesions are characterized by a mix of high and low densities, with areas of low density corresponding to necrosis and high density indicating intratumoral bleeding on non-contrast-enhanced CT. Most enhanced CT scans show marginal or moderate enhancement although nodular enhancement may also be seen (15). Some typical PSAs exhibit hemangioma-like enhancement, with the solid portion of the tumor showing lamellar or central centrifugal enhancement, while cystic necrotic areas remain unenhanced. MRI findings include single, multiple, or diffuse round T1 signals of varying sizes, with longer or shorter T2 signals and small flaky hyperintensities on T2WI when tumor necrosis is present. On diffusion-weighted imaging (DWI), the lesion margins show uneven nodular hyperintensity, and apparent diffusion coefficient (ADC) maps show corresponding areas of low signal. Enhanced MRI scans reveal mild or marked uneven annular enhancement in the arterial phase, with progressive enhancement in the portal and delayed phases, and no enhancement in the areas of internal bleeding or necrosis. However, traditional imaging methods have limited specificity and ability to differentiate common spleen diseases (16). ^18^F-FDG PET/CT, a functional and metabolic imaging technique, is useful for the diagnosis and staging of PSA. It allows for a whole-body assessment in a single imaging session, detecting lesions in sites other than the spleen. ^18^F-FDG PET/CT findings in PSA are mainly characterized by diffuse and unevenly increased uptake of ^18^F-FDG in the spleen, with an abnormal rise in SUVmax. A review of related cases (**Table 1**) shows that SUVmax values for PSA range from 3.89 to 19, with a median of 10.1 (17). In this case, there was an abnormally increased, diffuse, and uneven uptake of ^18^F-FDG in the spleen, with some areas showing circular high uptake (SUVmax 9.0), and no ^18^F-FDG uptake in the necrotic areas. Additionally, there was an abnormally increased focal uptake of ^18^F-FDG in the liver (SUVmax 5.6), with the corresponding nodular low-density shadows on CT images, suggesting metastatic involvement. Therefore, for solid splenic masses, ^18^F-FDG PET/CT can non-invasively differentiate between benign and malignant lesions, and it can also identify systemic metastases in the omentum, bone, lungs, lymph nodes, and other sites. ^18^F-FDG PET/CT not only provides more accurate staging but also guides the biopsy site selection for lesion puncture. The integration of clinical symptoms and related examinations offers diagnostic insights for clinical decision-making and has a high negative predictive value (18).

**Table 1:** Literature reports of PSA of ^18^F-FDG PET/CT in a foreign language in recent years.

Author	Age (years)	Gender	Sign of ^18^F-FDG PET/CT	Metastasis	Survival at the time of report
SUN Ting	64	Female	Solid cystic mass in the spleen, increased FDG metabolism in the solid part and an SUVmax of 19.0, no uptake in the necrotic area	Chest wall, ribs	1 month post-operation, follow-up indicated that this patient recovered well without evident recurrence of tumor
Chen HT	53	Female	The diffuse uneven uptake of FDG was abnormally increased, with some annular high uptake and an SUVmax of 9.0	Bone	Without reporting
Iris Dirven	70	Female	Hypermetabolism at the edges of the splenic lesion	None	Survival after surgery 4 years
Iris Dirven	36	Female	Hypermetabolism in both the spleen and liver	Liver	After several months, the patient was lost to follow-up
Iris Dirven	75	Female	Hypermetabolic lesions in the liver, spleen, and spine	Liver, spleen, spine	Died within 2 weeks of the diagnosis and 7 months after the initial presentation
Zhao Qian	44	Male	Increased non-uniform FDG uptake with an SUVmax of 11.6 of the entire spleen	Liver	Eight months after surgery
Zheng, Kun	43	Female	The masses and nodules of the spleen had a mildly increased uptake of FDG	None	Without reporting
Abe, Takashi	53	Male	A solitary splenic tumor of 140 mm invading the liver and left diaphragm. FDG accumulated in the periphery of the tumor.	Liver, left diaphragm	Five months after the operation
BD Nguyen	54	Male	A heterogeneous “polka-dot” FDG uptake features representing the malignant foci of tumor proliferation throughout the splenomegaly	None	Without reporting
Barış Özcan	65	Female	The PET CT scan after surgery did not show any sign of metastasis.	None	Survival after surgery 5 months
Hiroaki Takahashi	62	Male	Diffuse FDG accumulation in the enlarged spleen (SUVmax of 3.89), the vertebral bodies, sacroiliac bone, humerus, femur, and clavicles	Bone marrow	The patient died 17 weeks from the onset
Atsushi Tomioka	37	Male	A low attenuation solid mass protruding from the lower pole of the spleen. A cystic component with mural calcifications adjacent to the mass was shown. The tumor was FDG-avid.	None	Ten years have passed since the patient’s splenectomy and he continues to do well, without evidence of local or distant recurrence

PSA must be differentiated from other splenic tumors, such as splenic hemangioma, splenic lymphoma, and splenic metastases. Splenic primary malignant lymphoma is the most common primary malignant tumor of the spleen, predominantly of the diffuse large B-cell type. Its pathological classification influences its imaging manifestations, which can include diffuse infiltrating, miliary, multiple nodules and large mass types. On enhanced CT images, it often presents as multiple or large low-density shadows, and on ^18^F-FDG PET/CT, it typically shows intense and uniform FDG uptake (19). In addition, the presence of lymphomatous lymph node involvement and the detection of common sites of metastasis can also help distinguish between lymphoma and PSA. Splenic hemangiomas are benign tumors and are typically not FDG-avid on ^18^F-FDG PET/CT, making them relatively easy to differentiate. Splenic metastases, which are often associated with a history of a primary tumor, can be identified using ^18^F-FDG PET/CT. This imaging modality can help distinguish between these various conditions by evaluating the pattern and intensity of FDG uptake, as well as by identifying any secondary sites of disease involvement.

PSA is a highly malignant tumor that tends to metastasize early, most frequently to the liver, lungs, lymph nodes, and bone. In this case, the patient exhibited decreased red blood cell count, elevated white blood cell count, reduced platelets, and an increase in CA125 as a tumor marker, while other laboratory tests were within normal limits. Early diagnosis is challenging, and the definitive diagnosis relies on pathology. Given that PSA typically presents with splenomegaly, heterogeneous density within the mass, a propensity for necrosis, and a high risk of splenic rupture, the primary treatment for PSA involves surgical resection of the spleen, followed by postoperative radiotherapy and chemotherapy. However, the prognosis remains poor (20). Takehara M et al. (21) reported that the 6-month survival rate for patients with PSA is only 20%, with rare instances of survival exceeding 2 years. Therefore, early diagnosis is crucial for the effective treatment and improved prognosis of splenic angiosarcoma.

## Conclusions

In summary, PSA is a rare condition characterized by non-specific clinical and radiological features. A confirmed diagnosis relies on pathological findings, with immunohistochemical analysis playing a pivotal role in the diagnostic process. PSA should be considered when a space-occupying lesion in the spleen is detected, especially if it presents with necrotic areas, exhibits progressive enhancement on contrast-enhanced imaging, and shows heterogeneously increased FDG uptake. ^18^F-FDG PET/CT imaging offers valuable diagnostic insights into PSA and is particularly useful for preoperative staging, guiding biopsy procedures, assessing the response to postoperative treatment, and monitoring patients throughout their follow-up period.

## Data availability statement

The original contributions presented in the study are included in the article/supplementary material. Further inquiries can be directed to the corresponding author.

## Ethics statement

Ethical approval was not required for the study involving humans in accordance with the local legislation and institutional requirements. Written informed consent to participate in this study was not required from the participants or the participants’ legal guardians/next of kin in accordance with the national legislation and the institutional requirements. Written informed consent was obtained from the individual(s) for the publication of any potentially identifiable images or data included in this article.

## Author contributions

MS: Writing – review & editing, Writing – original draft, Software, Methodology, Data curation. WQ: Writing – original draft, Software, Data curation. RX: Writing – review & editing, Software, Data curation. ZL: Writing – review & editing, Software, Data curation. FL: Writing – review & editing, Data curation. SF: Writing – review & editing, Software, Data curation.
